# Dexamethasone-induced Intra-Uterine Growth Restriction impacts NOSTRIN and its downstream effector genes in the rat mesometrial uterus

**DOI:** 10.1038/s41598-018-26590-3

**Published:** 2018-05-29

**Authors:** Shreeta Chakraborty, Safirul Islam, Sarbani Saha, Rupasri Ain

**Affiliations:** Division of Cell Biology and Physiology, CSIR-Indian Institute of Chemical Biology, 4, Raja S.C. Mullick Road, Kolkata, West Bengal 700032 India

## Abstract

Intra-Uterine Growth Restriction (IUGR) is a major cause of fetal and neonatal mortality. Understanding the impact of IUGR on utero-placental gene expression is key to developing effective therapy. In this report we elucidated the impact of IUGR on NOSTRIN and its downstream effector gene expression in the utero-placental compartments. We showed here that induction of IUGR by maternal dexamethasone administration in rats led to up-regulation of NOSTRIN transcript and protein in the mesometrial triangle of the uterus (MG) and not in other utero-placental compartments as compared to control. This was associated with down-regulation of twelve genes and four cytokines that were known to be regulated by NOSTRIN and also required for maintenance of pregnancy. Interestingly, there was remarkable decrease in phosphorylation of RelA transcription factor in the MG during IUGR in line with the fact that the down regulated genes harbour RelA transcription activation domain in their promoters. Furthermore, HIF-1α level was reciprocal to NOSTRIN expression pattern in the mesometrial compartment during IUGR and also in CoCl_2_ treated endothelial cells. Over-expression of HIF-1α led to a decrease in NOSTRIN levels suggesting inhibition of *Nostrin* transcription by HIF-1α. Our findings highlight the importance of NOSTRIN in uterine pathophysiology during IUGR.

## Introduction

Mammalian placentation is associated with extensive growth and remodelling of maternal uterine vasculature. Improper development of maternal vasculature affects fetal development leading to constrained fetal growth in utero, commonly referred as Intra-Uterine Growth Restriction (IUGR). IUGR is a major cause of fetal and neonatal morbidity and mortality^1,2^ affecting about 30 million newborns per year^3^ and is associated with an increased risk of still birth^4^. The resulting newborns also have an increased risk of cardiovascular disease^5^, high blood pressure, diabetes, neuro-developmental progress and other metabolic disorders^6^. It is therefore of high priority to diagnose and prevent IUGR that happens at the most vulnerable period of human life. Dexamethasone-induced IUGR model of rat has been previously used to understand various molecular effectors that might be involved in the manifestation of IUGR^7–11^.

Maternal administration of Dexamethasone, a synthetic glucocorticoid, is being used for over four decades for fetal lung maturation in women at risk of preterm birth^12,13^. Placental 11-β hydroxysteroid dehydrogenase (11-β-HSD) protects the fetus from corticosteroid exposure by converting physiological glucocorticoids into inactive products. Since dexamethasone cannot be metabolised by 11-β-HSD, the negative side effects of dexamethasone administration might be severe in certain instances. Exposure of glucorticoid/dexamethasone retards fetal growth in animal models as well as in humans^14,15^. Dexamethasone-induced rat model of IUGR is well established^7–11^. However, the alteration of gene expression associated with the growth inhibitory effect of dexamethasone remains inadequately explored.

Several studies have shown that compromised angiogenesis at the utero-placental interface is often associated with pregnancies complicated by IUGR. Endothelial cell dysfunction and imbalance in expression profile of various angiogenic factors, such as, vascular endothelial growth factor, placenta growth factor and angiopoietin lead to restricted blood flow and further contributes to elevated fetoplacental vascular resistance^16^. Metrial gland, located in the mesometrial uterus, right above the placenta, is the uterine entry point of blood vessels supplying each placenta and fetus in rodents^17^. It can therefore, be predicted that vascular reactivity in the metrial gland is of paramount importance for appropriate development of fetus. However, there is lacuna in understanding the humoral effects of metrial gland derived molecules during pregnancy as well as in IUGR.

NOSTRIN (Nitric Oxide Synthase Trafficking INducer) was characterized as an eNOS-sequestering protein that altered its sub-cellular localization and prevented calcium ionophore-induced NO production^18,19^. NOSTRIN protein levels were found to be significantly up regulated in placentas of women with pre-eclampsia (PE)^20^ and pregnancy-induced hypertension (PIH) with a concomitant reduction in NO production^21^. A significant negative correlation between the expression of NOSTRIN and activity of eNOS was also demonstrated in umbilical vessels of women with PE^22^. Research from our laboratory showed that NOSTRIN is a pleiotropic functional modulator of endothelial cells^23^. Apart from its well-known function of sequestering eNOS and attenuating NO production, NOSTRIN can affect the functional transcriptome of endothelial cells by down-regulation of several angiogenesis promoting genes, independant of eNOS activity. Interstingly, NOSTRIN being an adaptor protein can directly bind to TRAF6 and suppress NFκB signalling in endothelial cells. As a consequence, there is also a decline in secreted factors such as cytokines and chemokines^23^.

Our interest in finding out the impact of IUGR in uterine gene expression and the compelling expression pattern of NOSTRIN during IUGR prompted us to explore the gene network known to be affected by NOSTRIN. Furthermore, we elucidated the role of hypoxia inducible factor-1α (HIF-1α) in regulating NOSTRIN expression due to presence of putative HIF-1α binding sites in NOSTRIN promoter. Data from these experiments highlight the overt significance yet unexplored function of NOSTRIN in uterine patho-physiology during IUGR.

## Results

### Induction of IUGR by maternal dexamethasone administration leads to up regulation of NOSTRIN in the mesometrial triangle of the uterus

**Figure 1:**
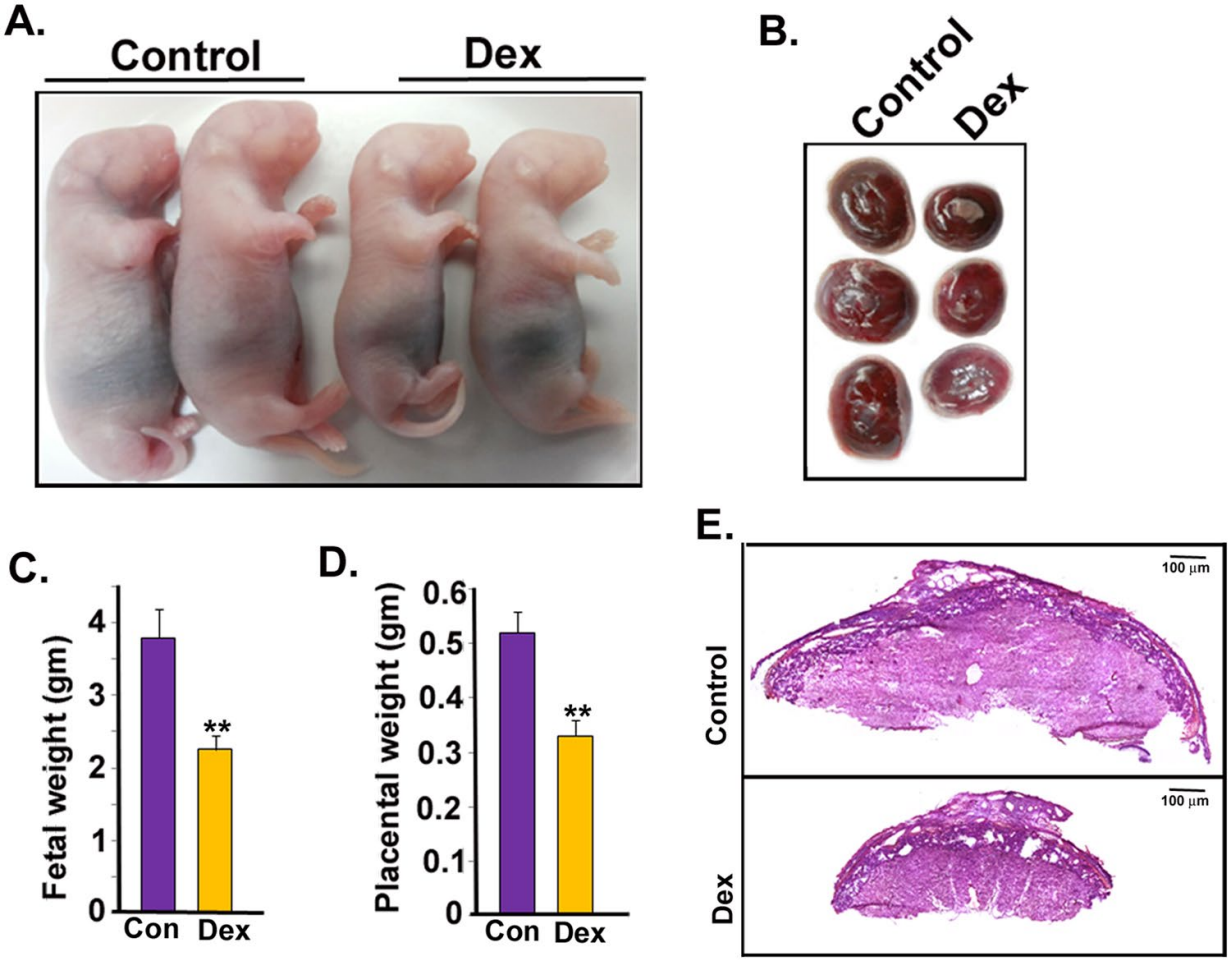
Induction of IUGR upon maternal administration of Dexamethasone acetate (Dex). Fetuses and placentas were collected on Day 20.5 of gestation. (**A**) Representative photographs of fetuses showing reduction in fetal size after Dex-treatment compared to Control (vehicle treated). (**B**) Diminution in placental size by Dex-treatment. Fetal and placental size was observed in 5 different biological replicates. (**C**) Fetal weight reduction occurred by 41% in Dex-treated group of rats as compared to Control group. (**D**) Placental weight decreased by 37% from the same group of rats used in C. (**E**) Eosin and Hematoxylin staining of mesometrialuteroplacental tissue from Control and Dex-treated rats on gestation Day 20.5 depicting various utero-placental zones. Error bars in C and D represent standard error of mean (SEM) from five biological replicates. (n = 5, **p < 0.01).

Dexamethasone administration during last third of gestation led to induction of IUGR, evident from an overall reduction in fetal (Fig. 1A) and placental size (Fig. 1B) accompanied by a 41% decrease in fetal weight (Fig. 1C) and 37% reduction in placental weight (Fig. 1D). There was significant (p < 0.01) reduction in fetal and placental weight on administration of dexamethasone acetate in 5 different biological replicates. However, dexamethasone treatment did not affect fetal viability or number of fetuses. Haematoxylin and eosin staining of placental section from control and IUGR-induced placenta (Fig. 1E) showed that there was no morphological abnormality of the placenta upon dexamethasone administration. This data is in agreement with previously published reports of maternal dexamethasone administration.

**Figure 2:**
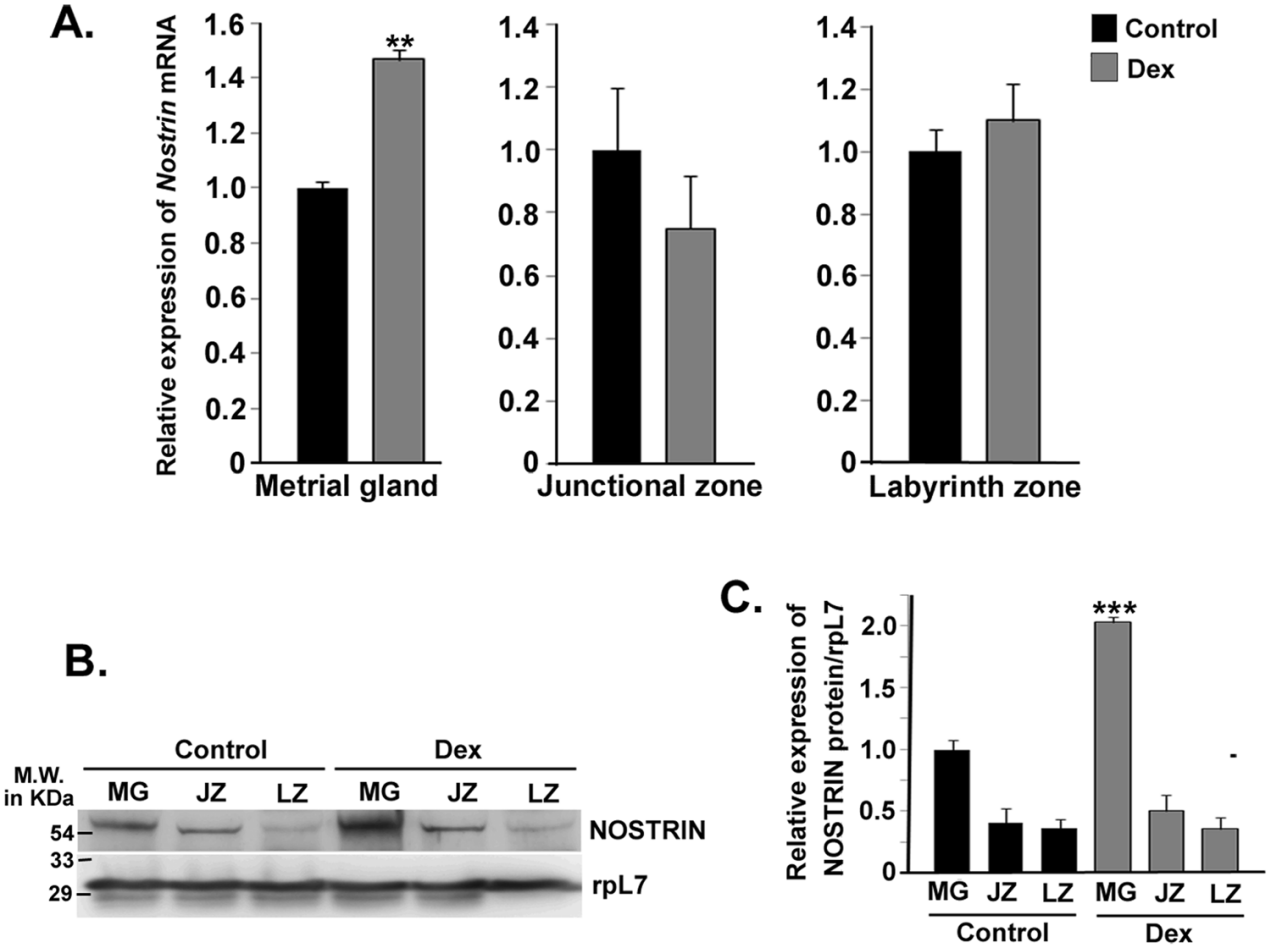
Effect of Dexamethasone (Dex) induced IUGR on NOSTRIN expression at gestation Day 20.5 in utero-placental tissues. (**A**) Quantitative real time PCR analysis of *Nostrin* transcript using RNA from three distinct utero-placental zones, Metrial gland (MG), Junctional zone (JZ) and Labyrinth zone (LZ) ofDex-treated and Control animals. The transcript levels were normalized relative to *Rpl7*. Experiments were repeated using five independent biological replicates. Error bars represent standard errors of mean. (**B**) Western blot analysis of NOSTRIN using the same tissue samples as described in (**A**). RpL7 was used as a loading control. Experiments were repeated using five independent biological replicates. (**C**) Quantification of NOSTRIN levels in Control and Dex treatment (from B) normalized to the loading control using ImageJ software. A significant (p < 0.005) up-regulation in NOSTRIN was observed in the metrialgland. Values are represented as mean ± SEM of three independent experiments. (n = 3, **p < 0.01,***p < 0.005).

Recent reports from our laboratory established the eNOS-independent function of NOSTRIN *in vitro*. We therefore, sought to analyze NOSTRIN expression in the context of utero-placental development in this rat model of IUGR. To analyze placental response to maternal dexamethasone treatment, *Nostrin* mRNA expression was assessed in various utero-placental compartments on gestation day 20.5 by quantitative real time PCR. Interestingly, there was no significant change in *Nostrin* mRNA levels in the junctional and labyrinth zones of the placenta. In contrast, there was remarkable (p < 0.01) up-regulation of NOSTRIN transcripts in the mesometrial compartment of the uterus, i.e., metrial gland (Fig. 2A). NOSTRIN protein levels were then monitored by immunoblot assay (Fig. 2B). Concordant to mRNA levels, expression of NOSTRIN was not substantially altered in the junctional and labyrinth zone of the placenta with respect to the control groups. However, there was a significant (p < 0.005) up-regulation in NOSTRIN protein levels in the metrial gland. Protein levels were also quantified after normalisation with rpL7 as shown in Fig. 2C.

### NOSTRIN levels are not altered by Dexamethasone treatment *in vitro*

**Figure 3:**
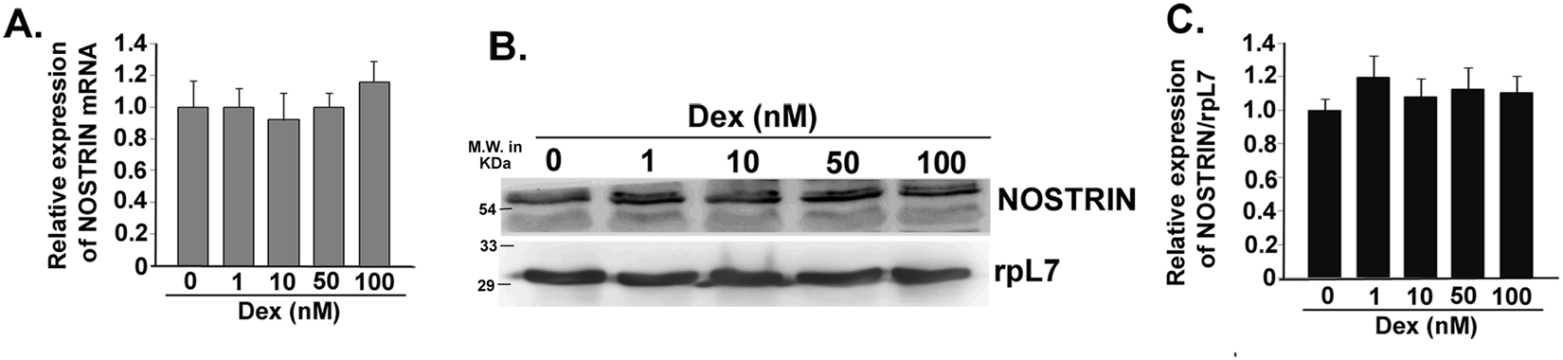
Dexamethasone treatment does not influence NOSTRIN expression *in vitro*. (**A**) Quantitative real time PCR analysis of *Nostrin* transcript in endothelial cells treated with increasing concentrations of Dexamethasone acetate (1 nM, 10 nM, 50 nM and 100 nM) compared to Control (treated with vehicle only). There was no significant change in mRNA levels. (**B**) Western blot analysis of NOSTRIN in endothelial cells treated with Dexamethasone acetate. RpL7 was used as a loading control and three different biological replicates were used. (**C**) Quantification of NOSTRIN protein (from B) normalized to the rpL7 using ImageJ software depicted no significant change. (n = 3, Not significant).

Although our data clearly demonstrated that NOSTRIN levels increased during IUGR, it still remained elusive whether this up-regulation was an influence of dexamethasone treatment or a consequence of IUGR. To address this question, we analysed whether dexamethasone treatment in endothelial cells affects NOSTRIN expression. MS1 endothelial cells were treated with increasing concentrations of dexamethasone. There was no significant change in *Nostrin* mRNA levels (Fig. 3A) determined by real time PCR or NOSTRIN protein levels (Fig. 3B) assessed by western blotting upon dexamethasone treatment even at highest concentrations of dexamethasone used. NOSTRIN protein levels were quantified and normalised to rpL7 levels (Fig. 3C). This data indicates that elevated level of NOSTRIN in the mesometrial triangle of the uterus is a consequence of IUGR induction.

### IUGR impairs uterine expression of NOSTRIN-regulated genes

**Figure 4:**
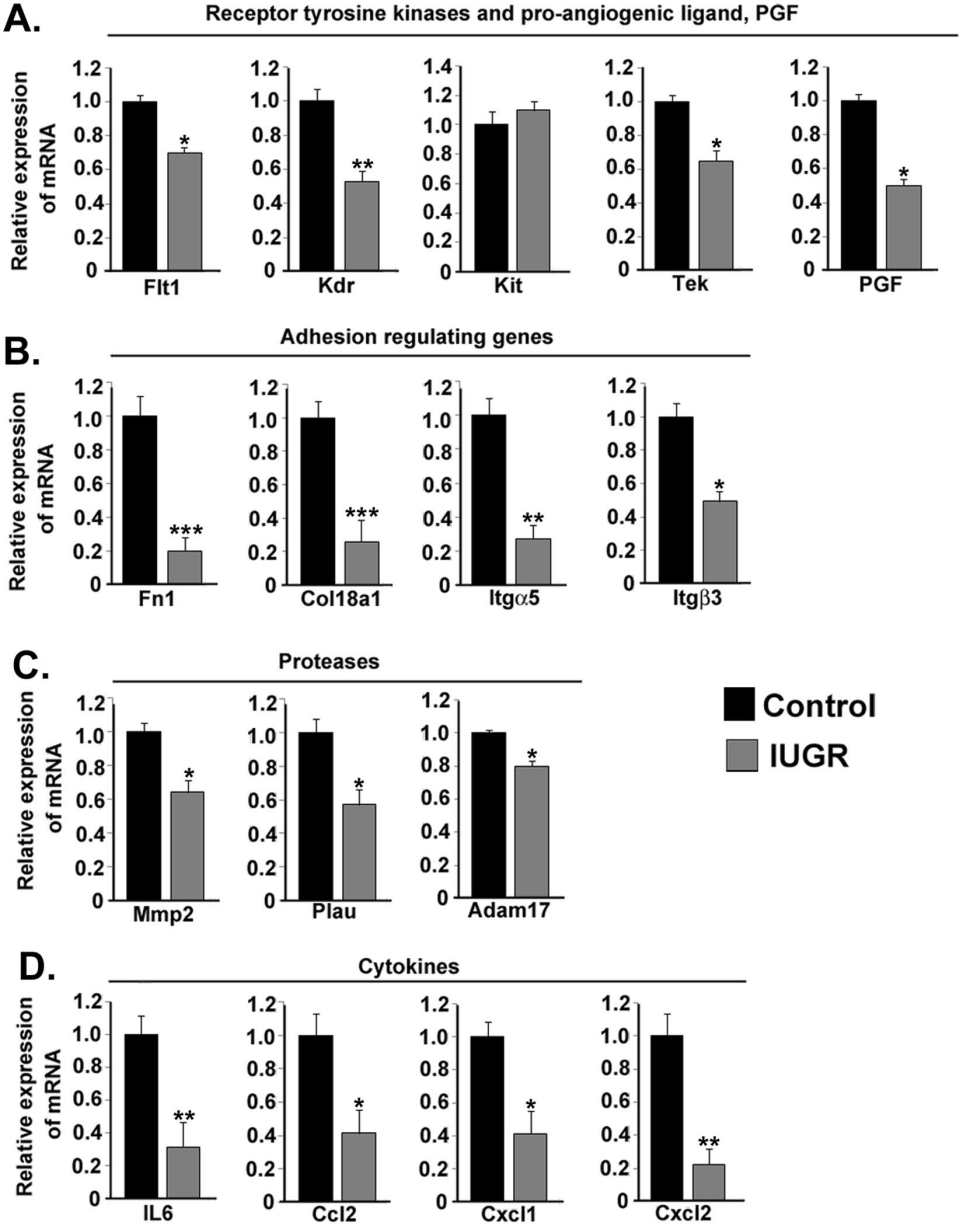
Induction of IUGR impairs metrial gland expression of genes that are known to be regulated by NOSTRIN. Real time PCR analysis of transcripts in the metrial gland. The amount of a specific mRNA was normalized relative to the amount of rpL7 (∆Ct = Ct_*gene*_ − Ct_rpL7_). Fold change of gene expression was measured by using 2^−∆∆Ct^, where ∆∆Ct denoted the change in ∆Ct values between Dex and control metrial glands RNA. Error bars represents standard error of mean from three different biological replicates. (**A**) Transcripts of the receptor tyrosine kinases including *Flt-1, Kdr, Tek*and the pro-angiogeneic ligand *Pgf* but not *Kit* was down regulated significantly in IUGR samples. (**B**) IUGR led to decrease in transcript levels of the genes involved in adhesion and invasion such as *Itgα5, Itgβ3, Fn1* and *Col18a1*. (**C**) Proteases, such as *Mmp2, Plau* and *Adam17* transcripts were decreased in IUGR metrial gland. (D) Pro-inflammatory cytokines such as *Il6, Ccl2, Cxcl1* and *Cxcl2* were down regulated in IUGR metrial glands. (n = 3, *p < 0.05, **p < 0.01 or ***p < 0.005).

Recent report from our laboratory showed that NOSTRIN negatively regulates several genesin endothelial cells^23^. Since NOSTRIN was significantly (p < 0.005) up-regulated in the metrial gland, we analyzed the expression of these NOSTRIN-regulated genes by real time PCR. Interestingly, these genes were found to be significantly (p < 0.05 or p < 0.01 or p < 0.005 as denoted in figure) affected during IUGR. These included the receptor tyrosine kinases, such as, *Flt-1*, *Kdr*, *Tek* and a pro-angiogenic ligand of *Flt-1*, i.e., *Pgf* (Fig. 4A); adhesion molecules, such as, *Itgα5*, *Itgβ3*, *Fn1* and *Col18a1* (Fig. 4B); and proteases, such as *Mmp2*, *Adam1*7 and *Plau* (Fig. 4C). Furthermore, NOSTRIN elevation was also associated with down regulation of several cytokines, such as, *IL6*, *Ccl2*, *Cxcl1* and *Cxcl2* (Fig. 4D) in the metrial gland.

### IUGR negatively impacts downstream effector proteins of NOSTRIN

**Figure 5:**
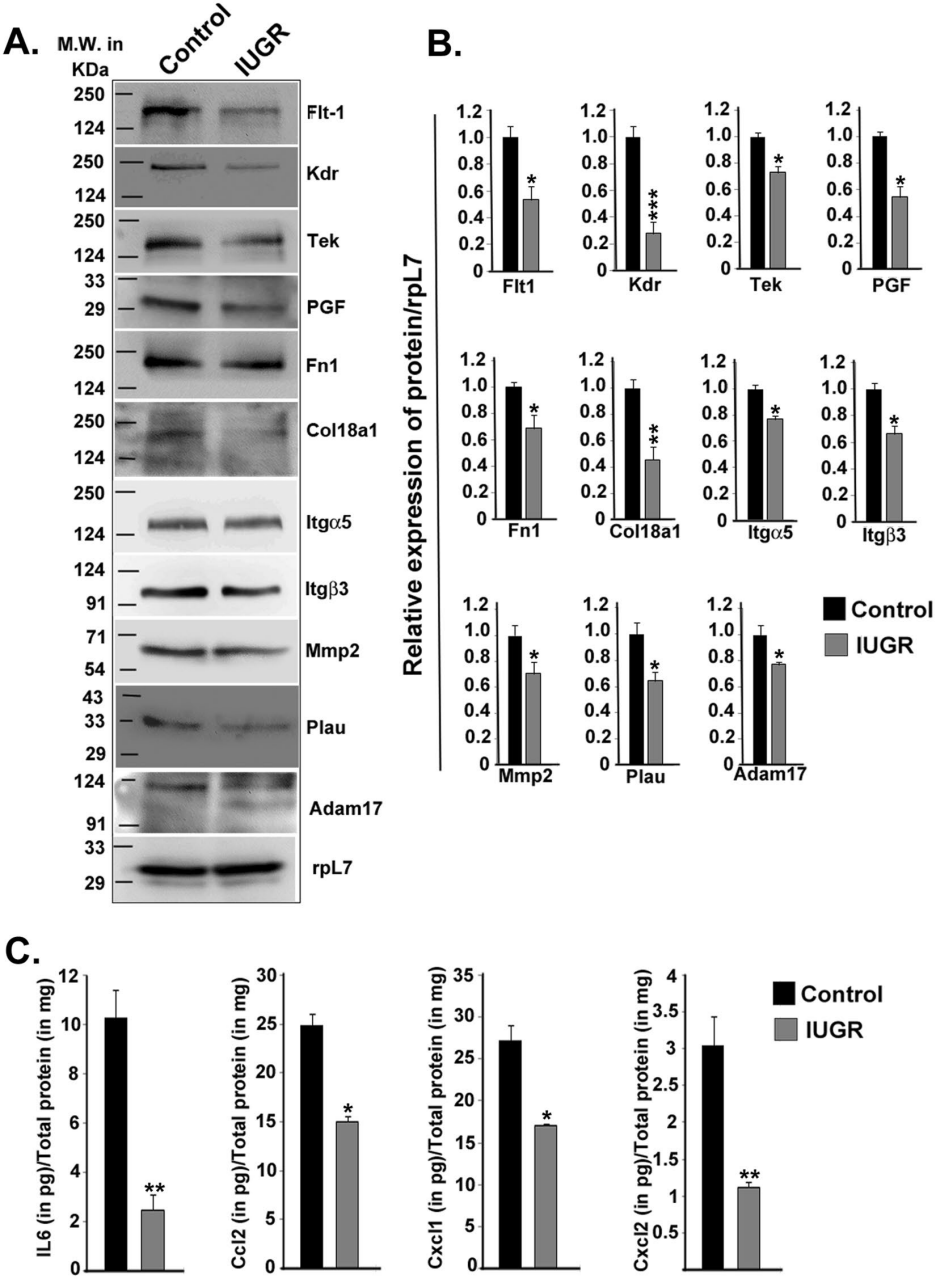
IUGR-induced NOSTRIN up-regulation was associated with decline in protein levels of NOSTRIN-regulated genes. (**A**) Immuno-blot analysis of proteins in the metrial gland in three different biological replicates. RpL7 was used as a loading control. Positions of the molecular weight markers for the spliced blots are denoted on the left side of the image. (**B**) Relative expression of the proteins (from A) was quantified after normalization with rpL7 using three biological replicates by ImageJ software. (**C**) ELISA analysis of secreted cytokines (IL6, CCL2, CXCL1 and CXCL2) during IUGR compared to Control. Cytokines were quantified in pg relative to total protein estimated in mg. ELISA experiments were repeated three times with different biological samples. Error bars represents standard error of mean. (n = 3, *p < 0.05, **p < 0.01 and ***p < 0.005).

To correlate the mRNA levels of affected genes with protein levels, either immunoblot assay or ELISA in case of cytokines was performed. All the genes that were down-regulated during IUGR reflected similar pattern of expression at protein levels also. Protein levels of receptor tyrosine kinases (FLT1, KDR, and TEK), pro-angiogenic ligand (PGF), adhesion promoting factors (Itgα5, Itgβ3, FN1 and COL18A1) along with proteases (MMP2, ADAM17 and PLAU) were assessed by immunoblot assay (Fig. 5A). Protein levels were quantified and normalised to rpL7 (Fig. 5B). All the aforesaid molecules were significantly (p < 0.05 or p < 0.01 as denoted in Fig. 5B) down-regulated by induction of IUGR in the mesometrial triangle of the uterus. There was also a significant decrease (p < 0.05 or p < 0.01 as denoted in Fig. 5C) in expression of secreted cytokines (IL6, CCL2, CXCL1 and CXCL2) as demonstrated by ELISA (Fig. 5C). These results highlight the importance of NOSTRIN and its associated proteins in the mesometrial uterus during IUGR.

### RelA phosphorylation is suppressed in the mesometrial uterus upon IUGR induction

**Figure 6:**
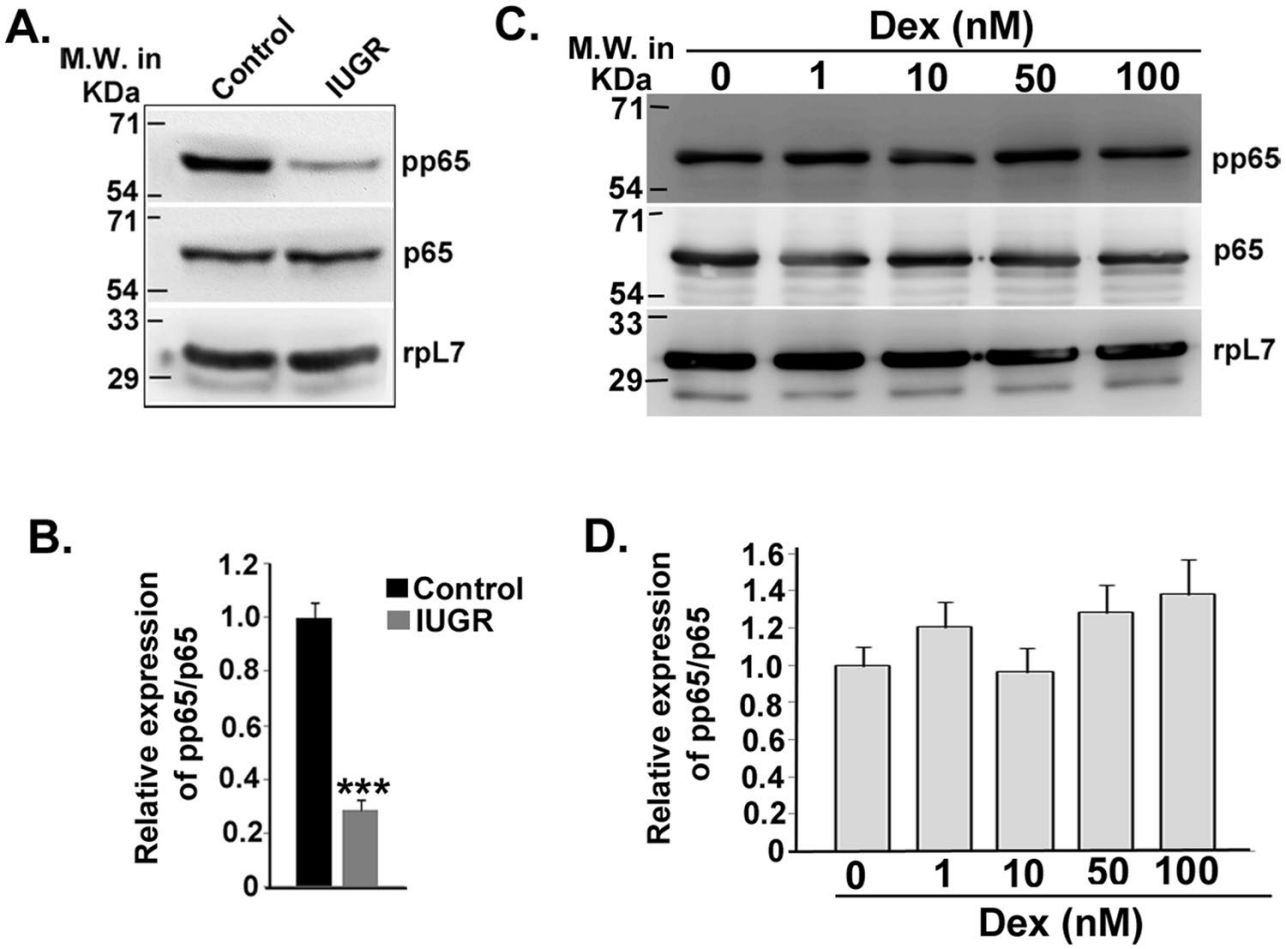
Suppression of RelA phosphorylation during IUGR (**A**) Western blot analysis of transcriptional nuclear factor κB (NFκB)/p65 and its phosphorylated form p-p65 at Ser-536 that leads to its trans-activation. Rpl7 is used as a loading control. (**B**) Relative expression of p-p65 with respect to p65 (from A) was quantified after normalization with rpL7 using three biological replicates from A by ImageJ software. (**C**) Immunoblot assay of phosphorylated p65 and p65 protein in endothelial cells treated with increasing concentrations of Dexamethasone acetate (1 nM, 10 nM, 50 nM and 100 nM) compared to Control (treated with vehicle only). RpL7 was used as a loading control. (**D**) Relative expression of p-p65 with respect to p65 (from C) was quantified after normalization with rpL7 using three biological replicates from C by ImageJ software. Error bars represents standard error of mean. (n = 3, For B, ***p < 0.005).

Recent data from our lab showed that NOSTRIN over-expression results in inhibition of NFκB signaling pathway^23^. Besides, promoter analysis and literature survey of the 11 genes along with the 4 cytokines that were affected during IUGR revealed that 9 out of 11 of the genes had NFκB response element. Cytokines IL6, CCL2, CXCL1 and CXCL2 have previously been demonstrated as direct targets of Rel transcription factors by chromatin immunoprecipitation assays. We, therefore, sought to investigate the phosphorylation status of RelAin the mesometrial uterus of IUGR rats. Nuclear factor κB (NF-κB)/Rel (p65) transcription factor having trans-activation domains are present in the cytosol in an inactive state, complexed with the inhibitory proteins. Phosphorylation of p65/RelA enhances its transcriptional activity. By immunoblot assay, we compared the protein levels of the p65/RelA and phosphorylated p65/RelA in uterine mesometrial triangle of control and IUGR rats. Interestingly, we found a significant (p < 0.005) decrease in the phosphorylation of p65 subunit in IUGR, whereas the total p65 levels remained unaltered as expected (Fig. 6A,B). Furthermore, we analyzed the levels of phosphorylated p65 in endothelial cells treated with dexamethasone by immunoblot assay (Fig. 6C) since corticosteroids are known to inhibit transcriptional activation of NFκB by various mechanisms^24,25^. There was no significant change in basal expression of p65 or activation of p65 by phosphorylation. Protein levels of pp65 were quantified and normalised to rpL7 levels (Fig. 6D). This data indicates that suppression of RelA phosphorylation in the uterine mesometrial triangle is not a consequence of dexamethasone treatment.

### NOSTRIN is negatively regulated by HIF1α in IUGR-induced mesometrial uterus

**Figure 7:**
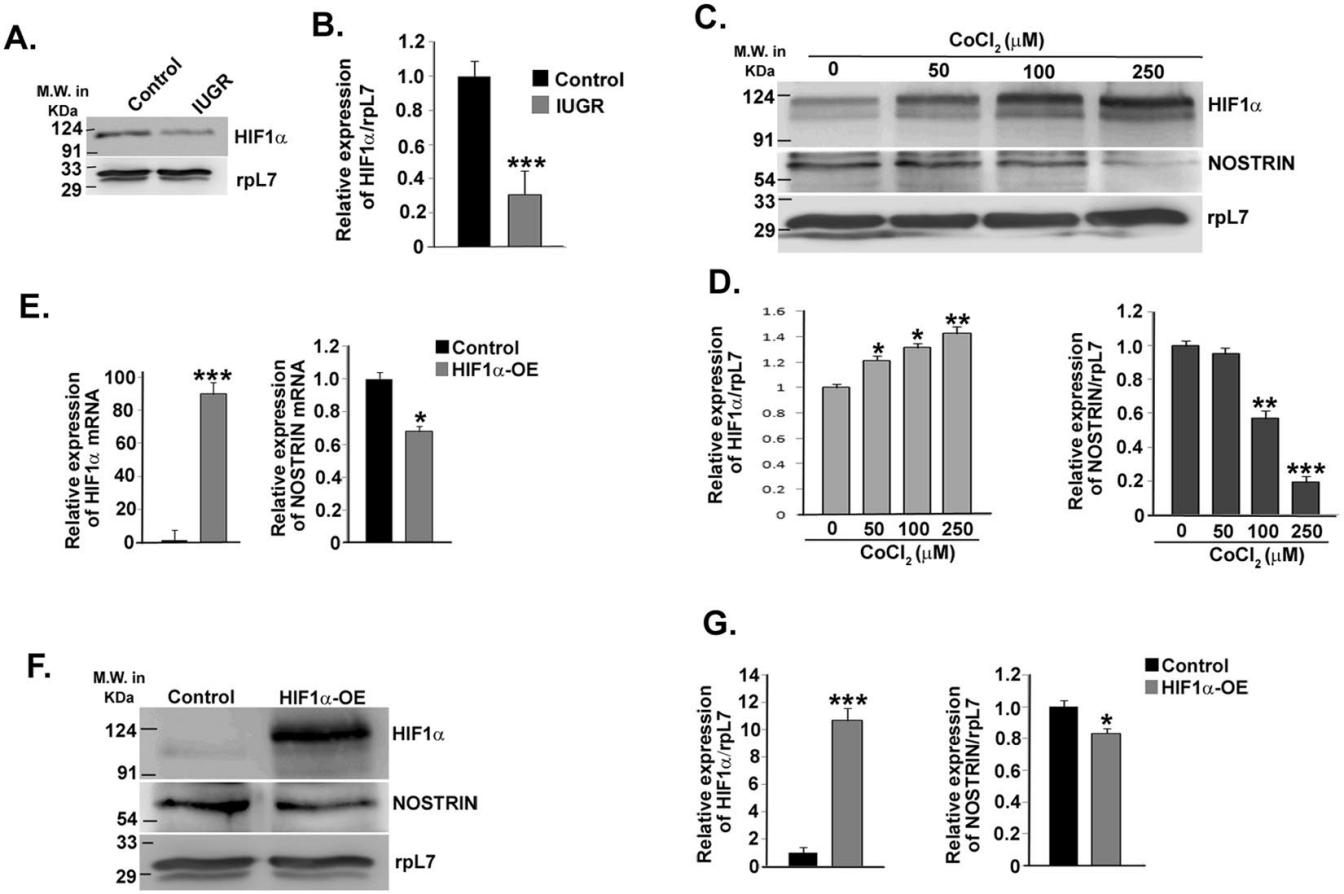
NOSTRIN is negatively regulated by HIF1α in the metrial gland and endothelial cells. (**A**) Western blot analysis of HIF-1α in the metrial gland during IUGR. RpL7 was used as a loading control. (**B**) Relative expression of HIF1α (from A) was quantified after normalization with rpL7 using three biological replicates by ImageJ software. (**C**) Immunoblot analysis of HIF-1α and NOSTRIN in endothelial cells treated with increasing concentrations of CoCl_2_. (**D**) Relative expression of NOSTRIN and HIF-1α levels (from C) were quantified after normalization with rpL7 using three biological replicates by ImageJ software. (**E**) Quantitative real time PCR analysis of *Hif-1α* and *Nostrin* transcripts in endothelial cells transfected with either empty vector or pCAG-HIF-1α. (**F**) Western blot analysis of HIF-1α and NOSTRIN in endothelial cells ectopically over-expressing HIF-1α. Empty vector transfected cells were used as control. (**G**) Relative expression of HIF-1α and NOSTRIN levels (from F) were quantified after normalization with rpL7 using three biological replicates by ImageJ software. Values for all quantification graphs are represented as mean ± SEM of three independent experiments.(n = 3, *p < 0.05, **p < 0.01 or ***p < 0.005 as denoted in figures).

Maternal hypoxia is known to be associated with development of IUGR^26^. Hypoxia inducible factor-1α (HIF-1α) is a master transcriptional regulator that is up-regulated under hypoxic conditions. Bioinformatic analysis showing the presence of hypoxia response element in NOSTRIN promoter indicates that HIF-1α may be a regulator of NOSTRIN transcription. We, therefore, analysed the protein levels of HIF-1α in the mesometrial compartment of dexamethasone induced IUGR by immunoblot assay and found a significant (p < 0.005) decline in HIF-1α levels by 70% (Fig. 7A) where NOSTRIN was up-regulated (See Fig. 2B). HIF-1α protein levels were also quantified and normalised to rpL7 (Fig. 7B). This reciprocal expression pattern of HIF-1α and NOSTRIN *in vivo* prompted us to induce and mimic hypoxia by CoCl_2_ treatment in endothelial cells. Interestingly, there was a dose dependant decrease in NOSTRIN levels on gradual induction of HIF-1α (Fig. 7C). Both HIF-1α and NOSTRIN protein levels were normalised to rpL7 (Fig. 7D). In order to show the specificity of HIF-1α mediated down regulation of NOSTRIN, we ectopically over-expressed HIF-1α in endothelial cells. Over-expression of HIF-1α in endothelial cells by transfection of pCAG-HIF-1α was analyzed by real time PCR and up-regulation in *Hif-1α* transcript (p < 0.005) was analyzed by real time PCR (Fig. 7E). Over-expression of HIF-1α led to 32% decline (p < 0.05) in *Nostrin* mRNA levels (Fig. 7E). Concordant to mRNA levels there was significant decline (p < 0.05) in NOSTRIN protein levels by over-expression of HIF-1α (Fig. 7F). Protein levels were quantified and normalised to rpL7 (Fig. 7G). Taken together, these data indicate that HIF-1α negatively regulate *Nostrin* transcription.

## Discussion

Several genes known to be regulator of angiogenesis have been implicated in IUGR as compromised development of the utero-placental vascular network leads to placental insufficiency^27–30^. Our data, for the first time, show that gene network in the uterus located at the vicinity of the developing placenta also is affected by IUGR. Gene ontology analysis of transcripts in the mesometrial compartment of the utero-placental section shows enrichment of genes required for vasculature development highlighting the importance of the metrial gland in vascular remodelling^31^. Our data clearly demonstrated that elevated NOSTRIN levels in the mesometrial uterus were associated with a decline in expression of genes known to be down regulated by NOSTRIN *in vitro*^23^. This gene network primarily includes angiogenesis promoting genes. Among the genes that were affected in our study, members of the VEGF family were most prominent. VEGF family members are known for their regulation of vasculogenesis and angiogenesis in the developing placenta^32–34^. FLT-1/VEGFRI, KDR/VEGFRII and PGF were found be down-regulated on induction of IUGR by maternal administration of dexamethasone. Binding of VEGF proteins to their receptors induces receptor homodimerization or heterodimerization, which in turn activates receptor kinase activity leading to receptor autophosphorylation and downstream signaling. PGF is a ligand for FLT1 known to stimulate developmental angiogenesis^35^. Genetic studies in animal models clearly demonstrate the importance of VEGF signaling in vasculogenesis. Embryos lacking VEGF receptors are embryonic lethal and exhibit severe vascular defects^36–38^. Our data reveals that there is decline in VEGF receptors in the mesometrial uterus on induction of IUGR.

Other than VEGF receptors, emerging evidence suggests that the Angiopoietin/TIE2 signaling system is critical for development and maintenance of placental vasculature during pregnancy. Deletion of TEK/TIE2 is also embryonic lethal due to failure of the vasculature system to expand^39,40^. Our data demonstrated that NOSTRIN up-regulation was associated with a reduction in TEK levels in the mesometrial compartment reinforcing the importance of Angiopoietin/TIE2 signaling system in the utero-placental interface.

Cell adhesion molecules not only play a crucial role in invasion of trophoblast cells to the uterine wall, but also aids in degradation and remodelling of basement membrane and surrounding extracellular matrix (ECM) during proliferation of endothelial cells at the onset of angiogenesis. NOSTRIN mediated down-regulation of ITGβ3, ITGα5 and FN1 has been recently demonstrated^23^. During IUGR, there was a decrease in these adhesion molecules along with an increase in NOSTRIN levels in the metrial gland. Apart from these adhesion molecules COL18A1 which is a component of the ECM itself was reduced during IUGR. Our data highlight the importance of regulatory role of adhesion markers in metrial gland during IUGR.

Proteolytic enzymes are well known to promote neovascularisation by aiding in degradation of the basement membrane and the ECM leading to formation of new vessels. A decline in expression of ADAM17, MMP2 and PLAU were associated with up-regulation of NOSTRIN in the mesometrial compartment during IUGR. Several reports suggest that these proteases also potentiates cancer progression^41–43^ since they aid in angiogenesis. Our data extends the importance of protease in the pathogenesis of IUGR.

Altogether, our study suggests that there was a decline in several NOSTRIN-regulated genes that might contribute to functional restriction in vascular development, particularly affecting the metrial gland on maternal administration of dexamethasone, leading to IUGR. Interestingly, most of the genes that were found to be regulated in the mesometrial triangle of the uterus during IUGR possess NFκB binding element in their promoter. Phosphorylation of Ser-536 of p65/RelA leads to trans-activation by increased binding of CBP (CREB binding protein) and subsequent acetylation of p65^44,45^. Our data clearly depicts that RelA phosphorylation was suppressed during IUGR. These data are in line with our results on down-regulation of the genes and cytokines that include IL6, CCL2, CXCL1 and CXCL2 in the mesometrial uterus which are direct targets of RelA transcription factor. However, there are earlier reports of Th1 bias and increased levels of inflammatory cytokine in IUGR cases as compared to normal pregnancy^46–49^. These enhanced levels of proinflammatory cytokines are secreted primarily either by PBMC or by trophoblast cells of the placenta. Our results show decreased levels of proinflammatory cytokines in the metrial gland which is rich in vasculature and is the entry point of placental arteries. Our data, therefore, indicates that down regulation of these cytokines by tissue specific modulators might be a consequence or compensatory response to prevent the placental insufficiency during IUGR.

Vascular manifestations of IUGR are often attributed to clinical conditions that lead to hypoxia^50–53^. Hypoxia-inducible factor (HIF) is a transcription factor comprising of a constitutively active HIF-1β subunit and an oxygen-sensitive HIF1α subunit. HIF-1α levels are low under normal conditions as it is rapidly degraded whereas hypoxia leads to its accumulation. HIF-1α forms a heterodimer with HIF-1β and binds to promoters of several target genes to activate transcription. In our model of dexamethasone induced IUGR there was a reduction in HIF-1α levels in the metrial gland along with an increase in NOSTRIN. Thus our data suggests that the mesometrial compartment of the uterus is not susceptible to the effect of hypoxia, unlike other placental zones. This reciprocal expression pattern of HIF-1α and NOSTRIN in the mesometrial uterus along with the presence of Hypoxia-responsive elements (HREs) in NOSTRIN promoter (determined by in silico analysis) further prompted us to elucidate the negative regulation of NOSTRIN by HIF-1α in endothelial cells. Indeed, induction of hypoxia by CoCl_2_ treatment led to a concomitant dose dependant reduction in NOSTRIN levels. Furthermore, ectopic over-expression of HIF-1α in endothelial cells also led to diminution in NOSTRIN mRNA and protein. Altogether our data provides evidence that HIF-1a might be regulating NOSTRIN expression in the mesometrial uterus during IUGR. IUGR is primarily a consequence of placental insufficiency that leads to deregulation in cellular homeostasis. Hypoxia induces autophagy in various cell types^54^. Increased autophagy has been demonstrated in placenta during IUGR and in cytotrophoblasts cultured under hypoxic stress^55^. Autophagy might be an adaptive mechanism to promote cell survival under stressed conditions. Induction of hypoxia in endothelial cells in a time dependent manner was indeed associated with a gradual up-regulation in autophagy markers such as Beclin1 and LC3II (Figure S1). Anti-apoptotic Bcl2 levels also increase suggesting a state where cells try to prevent apoptosis by promoting autophagy. mTORC activity was not affected (Data not shown) precluding nutrient starvation. A vast majority of long lived or protein aggregates including organelles are degraded by autophagy^56^ under stress conditions to recycle cellular components and promote cell survival. Thus decresed NOSTRIN levels by induction of hypoxia might also be a consequence of increased autophagy in the endothelial cells.

**Figure 8:**
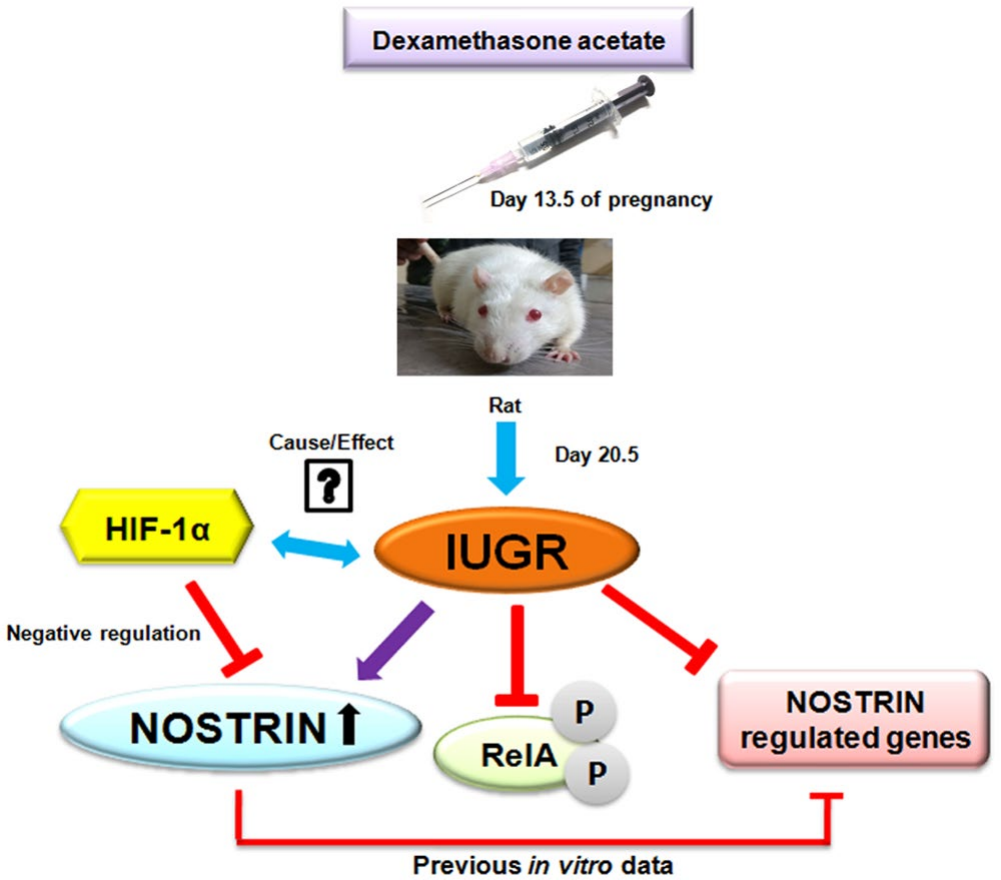
Effect of Dexamethasone-induced IUGR in the mesometrial uterus. Induction of IUGR by maternal administration of dexamethasone in the latter half of pregnancy in rat leads to increase in NOSTRIN levels associated with decline of RelA phosphorylation and diminished expression of genes known to be regulated by NOSTRIN. HIF-1α negatively regulates NOSTRIN expression. Taken together, these data highlight the importance of NOSTRIN in IUGR.

In summary, we demonstrate here a network of genes, known to be affected by NOSTRIN over expression *in vitro*, is down-regulated along with up regulation of NOSTRIN in the mesometrial uterus on induction of IUGR by dexamethasone treatment. Furthermore, there is remarkable curtailment of pro-inflammatory secreted cytokines such as IL6, CCL2, CXCL1 and CXCL2 in mesometrial triangle of the uterus during IUGR. In addition, RelA phosphorylation is suppressed during IUGR. Furthermore, HIF-1α, the master transcriptional regulator of hypoxia appears to negatively regulate NOSTRIN both *in vitro* and *in vivo*. A schematic representation of our findings is depicted in Fig. 8.

## Methods

### Animals and tissue preparation

Sprague Dawley rats (8–10 weeks old) were obtained Indian Institute of Chemical Biology (IICB) animal house. To obtain timed pregnancies, female rats were caged overnight with fertile males. Day 0.5 of pregnancy was denoted by the presence of sperm in morning vaginal smears. On Day 13.5 of pregnancy, 5 pregnant rats were subcutaneously injected with a bolus dose of 100 μg of Dexamethasone acetate (Sigma Aldrich, St. Louis, MO, USA) dissolved in 100 µl 10% ethanol. Control group of 5 pregnant rats were injected with vehicle only. Alzet osmotic pumps (Model 2 ML1, Durect Corp., Cupertino, CA, USA) were surgically implanted subcutaneously in the two groups of control and treated rats. The alzet pumps were pre-calibrated to release 200 μg of dexamethasone per kg of maternal body weight per day. Control group received osmotic pumps containing vehicle only. This model of IUGR in rats by dexamethasone treatment was previously established^7^. Animals were sacrificed on Day 20.5 of pregnancy. Placental and fetal weight was recorded for each of the rats. Utero-placental tissues were collected from control and dexamethasone treated rats and either snap-frozen in liquid nitrogen for RNA and protein analysis or frozen in dry-ice chilled heptane for haematoxylin and eosin staining. All tissue samples were stored at −80 °C until use.

Indian Institute of Chemical Biology Animal Ethics and Care Committee approved all procedures for handling and experimentation with rats as per guidelines set forward by the Committee for the Purpose of Control and Supervision of Experiments on Animals (CPCSEA), Govt. of India (http://cpcsea.nic.in).

### Cell culture and treatments

Mouse endothelial cell line, MS1 was obtained from American Type Culture Collection (Manassas, VA, USA). Endothelial cells were grown in Dulbecco’s Modified Eagle’s Medium, high glucose (Sigma Aldrich, St. Louis, MO, USA) supplemented with 5% FetalBovine Serum and Penicillin-Streptomycin (Invitrogen, Carlsbad, CA, USA) as per instructions from ATCC. Endothelial cells were treated with dexamethasone acetate dissolved in ethanol at final concentrations of 1 nM, 10 nM, 50 nM and 100 nM. Control cells were treated with vehicle only. For induction of hypoxia, endothelial cells were treated with CoCl_2_, (Sigma Aldrich, St. Louis, MO, USA) dissolved in DPBS (Invitrogen, Carlsbad, CA, USA) solution at final concentrations of 50 μM, 100 μM and 250 μM. Cells were incubated for 48 h before RNA or protein isolation.

### Cloning and transfection of HIF-1α

Mouse placental RNA was reverse-transcribed using superscript-III reverse transcriptase (Invitrogen, Carlsbad, CA, USA). Full length mouse HIF-1α (NM_001313919.1) cDNA was amplified from the placental cDNA using LA-Taq DNA polymerase (TaKaRa Bio, Mountain View, CA, USA). Primers used for full length HIF-1α were Fwd: 5′-TATAGGTACCGATGGAGGGCGCCGG-3′ and Rev: 5′-TAATGCGGCC GCTCAGTTAACTTGATCCAA-3′. Forward primer had KpnI and reverse primer had NotI restriction site at the 5′ end. The amplified cDNA was cloned in pCAG-DsRed vector (Addgene, Cambridge, MA, USA) by deleting dsRed. Transfection was performed using Lipofectamine LTX and Plus reagent (Invitrogen, Carlsbad, CA, USA). Control cells were transfected with empty vector backbone without dsRed and pCAG-HIF-1α expression vector was used for over-expression of HIF-1α in endothelial cells. Transfected cells were incubated for 48 h before RNA or protein isolation.

### RNA preparation and Quantitative real time PCR analysis

Total RNA was isolated from both tissues and cells using Trizol reagent (Invitrogen, Carlsbad, CA, USA). Five μg of total RNA was reverse transcribed using SuperScript III Reverse Transcription kit (Invitrogen, Carlsbad, CA, USA)according to the manufacturer’s instructions. Ten-fold dilution of cDNAs and Power SYBR GREEN PCR Master Mix (Applied Biosystems, Foster City, CA, USA) was used for each PCR reaction. Reactions were run using a 7500 Real-Time PCR System (Applied Biosystems, Foster City, CA, USA). Conditions used included initial holding stage (95 °C for 10 min) and 40 cycles (95 °C for 15 s and 60 °C for 1 min) followed by a dissociation stage (95 °C for 15 s, 60 °C for 1 min, and then 95 °C for 30 s). Primers used for real time PCR are enlisted in Table 1. Samples were normalised to housekeeping gene, rpL7 for each of the genes. At least three different biological replicates were used.

**Table 1:** Primer sequences used for real time PCR analysis.

Sl. No.	Primer name		Sequence (5′ to 3′)	Gene Bank Accession no.
1	rFlt1	Fwd	CGTGAAGCATCGGAAGCAA	NM_019306.2
		Rev	ACCGAATAGCGAGCAGATTTCT	
2	rKdr	Fwd	GCGGGAAACTACACGGTCAT	NM_013062.1
		Rev	CGTCTGCATGGTGCCATACT	
3	rKit	Fwd	TGACCAGATTGAAAGGCACAGA	NM_022264.1
		Rev	CACACTGGAGCCTGCCATT	
4	rTek	Fwd	TGTAGTGGATCAGAGGGATGCA	NM_001105737.1
		Rev	ACAGTGGCACCTGAGCTTACAG	
5	rPGF	Fwd	CTGCTGGGAACAACTCAACAGA	NM_053595.2
		Rev	GAAGGACACATGACGGACTGAA	
6	rFn1	Fwd	GGAAGTGTGAGCGACATGTTCTA	NM_019143.2
		Rev	GACCACACCGCTGTCTGTGA	
7	rCol18a1	Fwd	ACAAGTTCCAGGGAATGATCTCA	NM_053489.2
		Rev	GCCTGTGTAAATTGCTGCTTTCT	
8	rItgα5	Fwd	CATGTGTACCTGGGTGACAAGAA	NM_001108118.1
		Rev	GGAGAAGTTCCCTGGGTGTCT	
9	rItgβ3	Fwd	GACCACAGTGGGAGTCCTGTCT	NM_153720.1
		Rev	TGAGGCAGGTGGCATTGAAG	
10	rMmp2	Fwd	TCCCCTGATGCTGATACTGACA	NM_031054.2
		Rev	CCGCCAAATAAACCGATCCT	
11	rPlau	Fwd	GTGGTGGGAGCCTCATCAGT	NM_013085.3
		Rev	GCTGCTCCACCTCAAACTTCA	
12	rAdam17	Fwd	CGTTGGGTCTGTTCTGGTTTTC	NM_020306.2
		Rev	GCTGAGTCCATGCTGCTCAA	
13	rIL6	Fwd	TCACAGAGGATACCACCCACAA	NM_012589.2
		Rev	CTGACAGTGCATCATCGCTGTT	
14	rCcl2	Fwd	GGCCTGTTGTTCACAGTTGCT	NM_031530.1
		Rev	CCTGCTGCTGGTGATTCTCTT	
15	rCxcl1	Fwd	GATTCACTTCAAGAACATCCAGAG	NM_030845.1
		Rev	AGCATCTTTTGGACAATCTTCTGA	
16	rCxcl2	Fwd	CTACCAAGGGTTGACTTCAAGAA	NM_053647.1
		Rev	TTGGACGATCCTCTGAACCAA	
17	rRpl7	Fwd	GCCCTGAAGACACTGCGAAA	NM_001100534.1
		Rev	TGGTTCTGCGGGCACATAG	
18	rNOSTRIN	Fwd	ACACACCAAGTCCTGAGTATGCA	NM_001024260.1
		Rev	AAGGGCTTCATGCTTCTTCGT	
20	mNOSTRIN	Fwd	GCTTCTCCTGGCTGACTATTTTG	NM_181547.3
		Rev	CTTCCGCTCCAAGCCTTCTT	
22	mHIF-1α	Fwd	ATTTTGGCAGCGATGACACA	NM_001313919.1
		Rev	GGCTTTGGAGTTTCCGATGA	

### Western Blot analysis

Tissues were homogenised in RIPA buffer (20 mM TrisHCl, pH 7.5, 150 mM NaCl, 1 mM Na_2_ EDTA, 1 mM EGTA, 1% NP40, 1% sodium deoxycholate, 2.5 mM sodium pyrophosphate, 1 mM β-glycerophosphate, 0.2 mM PMSF, and 1 mM sodium orthovanadate) supplemented with protease inhibitor cocktail (Sigma Aldrich, St. Louis, MO, USA). For cellular protein extraction, cells were lysed in RIPA buffer followed by sonication (30 seconds per pulse, 3 pulses per sample at 10 MHz). Samples were then centrifuged and supernatants were collected. Protein concentration was determined using Bio-Rad Protein Assay Dye Reagent Concentrate (Bio-Rad, Hercules, CA, USA). The cell lysates were fractionated by SDS PAGE and transferred onto nitro-cellulose membrane. Blots were incubated for 1 h in blocking solution and overnight with primary antibodies. After washing, secondary antibody incubation was performed for 1.5 h at room temperature. An ECL reagent, Luminata Forte (Millipore, St. Charles, MO, USA) was used for chemiluminescence signal detection. Images were acquired with the Chemidoc Imaging System (UVP LLC, Upland, CA), USA) and band intensities were quantified with NIH ImageJ software.

### Enzyme-linked Immunosorbent Assay (ELISA)

Metrial gland tissues from control and IUGR rats were homogenised in tissue extraction buffer containing 1 mM Na_2_ EDTA, 1 mM EGTA, 2.5 mM sodium pyrophosphate, 1 mM β-glycerophosphate, 0.2 mM PMSF, and 1 mM sodium orthovanadate dissolved in DPBS (pH 7.4) and supplemented with protease inhibitor cocktail (Sigma Aldrich, St. Louis, MO, USA). Samples were centrifuged and supernatant was collected and stored at −80 °C until use. Expression of IL6, CCL2, CXCL1 and CXCL2 were determined using Rat IL6 Quantikine ELISA Kit (R6000B), Rat MCP1 Quantikine ELISA kit (MJE00), Rat CXCL1/CINC1 Quantikine ELISA Kit (RCN100) and Rat CXCL2/CINC3 Quantikine ELISA Kit (RCN300) respectively as per manufacturer’s instruction (R&D Systems, Minneapolis, MN USA).

### Antibodies

Anti-NOSTRIN (ab116374) antibody was purchased from Abcam (Cambridge, MA, USA) and was used in 1:100 dilutions. Other primary antibodies were purchased from either Cell Signaling Technology (CST, Beverly, MA, USA) and used in 1:1000 dilutions or from Santa Cruz Biotechnology (SCBT, Dallas, TX, USA) and used in 1:250 dilutions. Antibodies obtained from CST are as follows: anti-HIF1α (14179), anti-INTEGRIN α5 (98204), ANTI-INTEGRIN β3 (4702), anti-VEGF Receptor-2/KDR (9698), anti-BECLIN1 (3495), anti-LC3A/B (12741), anti-SQSTM1 (23214) and NF-κB Pathway Sampler Kit (9936). Others, procured from SCBT are anti-TACE/ADAM-17 (sc-6416), anti-COL18A1 (sc-16651), anti-FLT-1 (sc-316), anti-FIBRONECTIN (sc-6952), anti-MMP-2 (sc-10736), anti-PGF (sc-1883), anti-uPA (sc-14019), anti-TIE-2/TEK (sc-324), and anti BCL2 (sc-7382). HRP conjugated goat anti-rabbit and rabbit anti-mouse antibodies were purchased from Cell SignalingTechnology, and HRP conjugated donkey anti-goat antibodies were purchased from Santa Cruz Biotechnology. Both the secondary antibodies were used in 1:2000 dilutions.

### Statistical analysis

All experiments were performed using 5 different biological replicates and data were expressed as mean ± standard error of the mean (SEM). Statistical significance was assessed by the Student’s t-test. Two-sided p < 0.05 was considered significant for the mean values.

## Electronic supplementary material

Figure S1 and original blot imprints
