# Supplementary material for: Dexamethasone-induced Intra-Uterine Growth Restriction impacts NOSTRIN and its downstream effector genes in the rat mesometrial uterus
Source: Sci Rep. 2018 May 29;8:8342. doi: 10.1038/s41598-018-26590-3 (PMC5974239; doi:10.1038/s41598-018-26590-3)

# Dexamethasone-induced Intra-Uterine Growth Restriction impacts NOSTRIN and its downstream effector genes in the rat mesometrial uterus

Shreeta Chakraborty[1], Safirul Islam[1], Sarbani Saha[1] and Rupasri Ain[1,2]

[1]Division of Cell Biology and Physiology, CSIR-Indian Institute of Chemical Biology, 4, Raja S.C. Mullick Road, Kolkata 700032, West Bengal, India

[2]Address for correspondence: Dr.Rupasri Ain, Division of Cell Biology and Physiology, CSIR-Indian Institute of Chemical Biology, 4, Raja S.C. Mullick Road, Jadavpur, Kolkata 700032, West Bengal, India. Telephone: +91 (033) 24995876; Email: rupasri@iicb.res.in

**Figure S1**

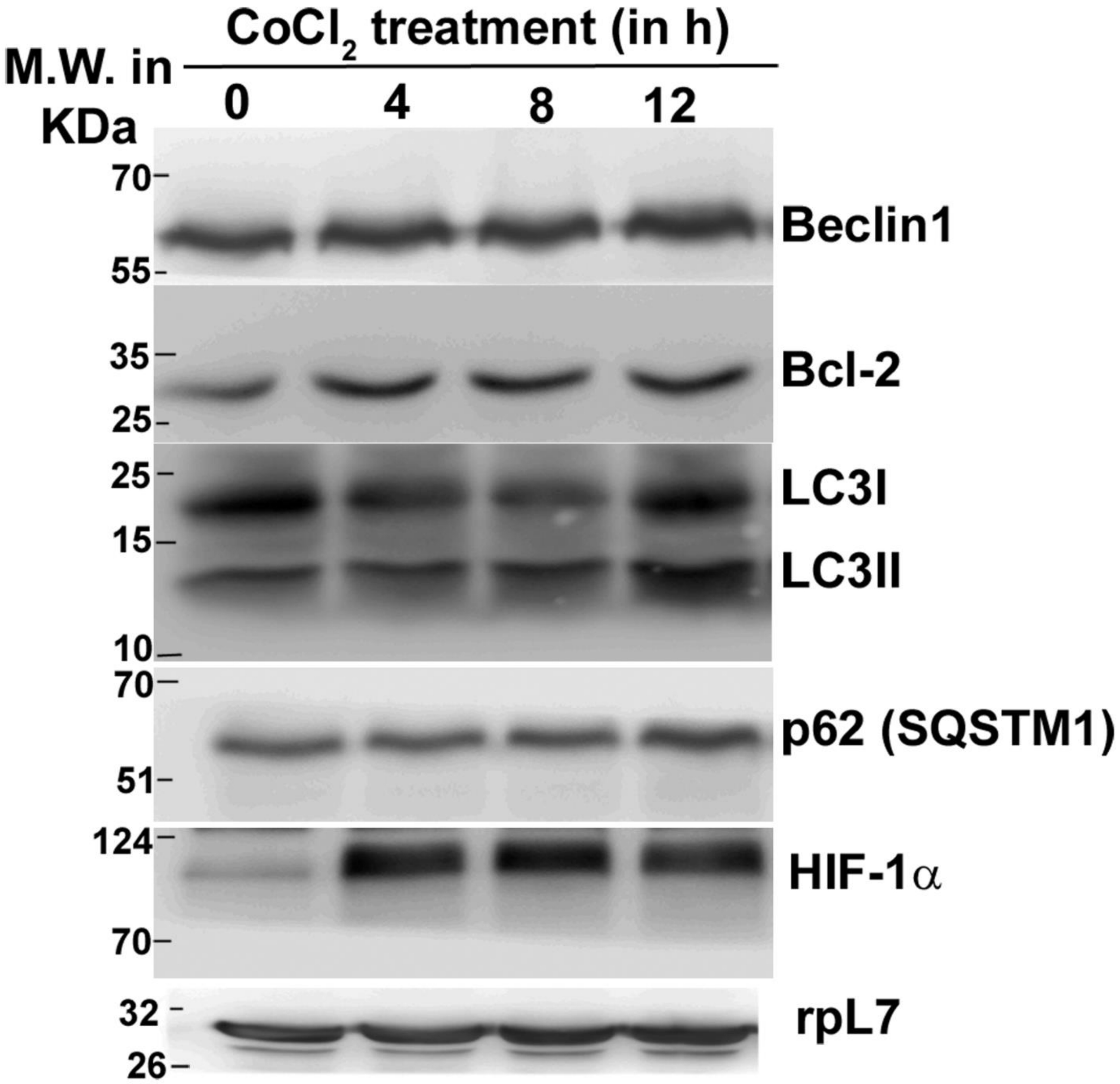


***Figure S1: Hypoxia-induced autophagy in endothelial cells.***

*Western blot analysis of autophagy markers (Beclin1, LC3 and p62) and anti-apoptotic protein (Bcl2) in a time dependent induction of hypoxia using protein from endothelial cells treated with 250 μm $CoCl_2$. rpL7 was used as a loading control.*

## ORIGINAL BLOTS:

Figure 2B

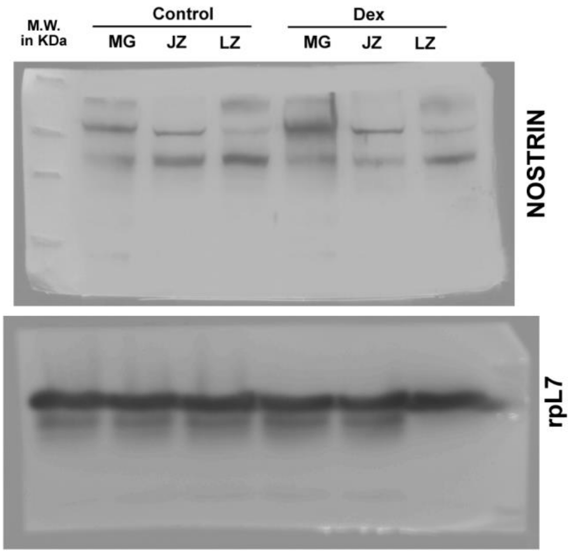


Figure 3B

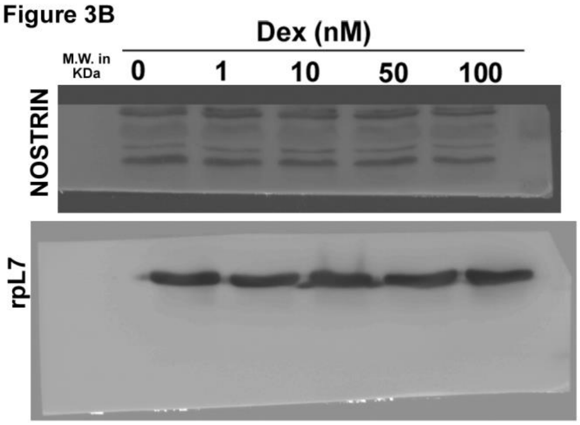

Figure 5A

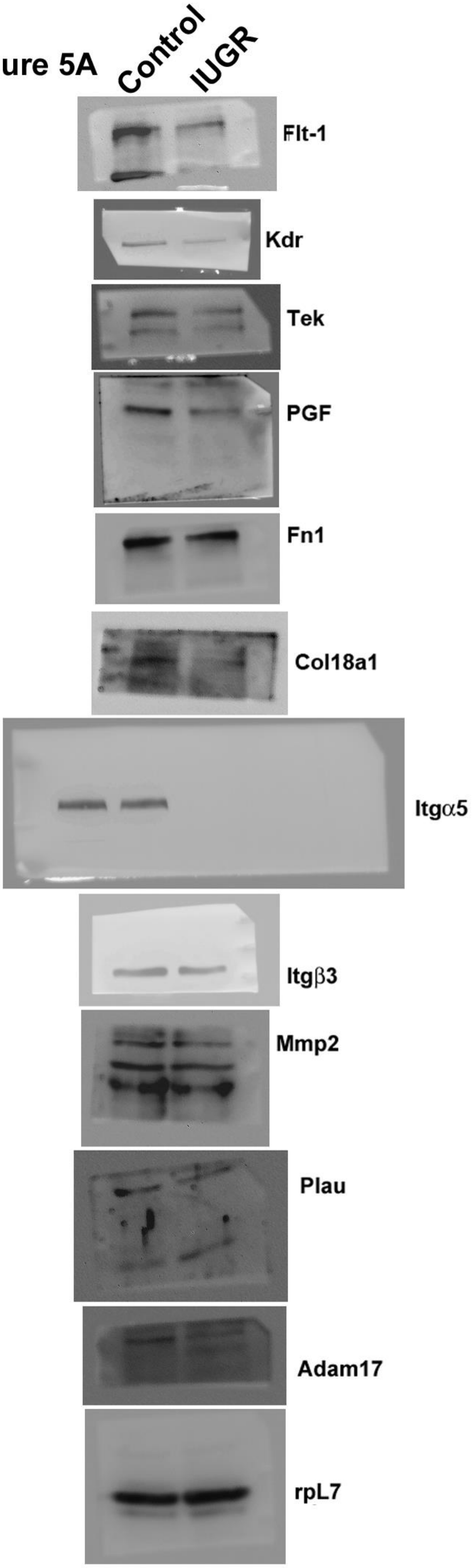

Control
IUGR
Flt-1
Kdr
Tek
PGF
Fn1
Col18a1
Itgα5
Itgβ3
Mmp2
Plau
Adam17
rpL7

Figure 6A

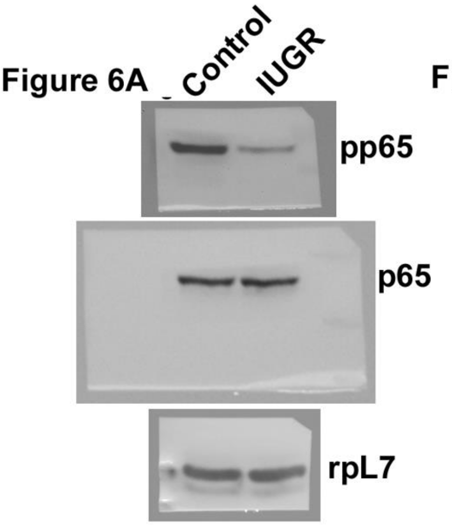


Figure 6C

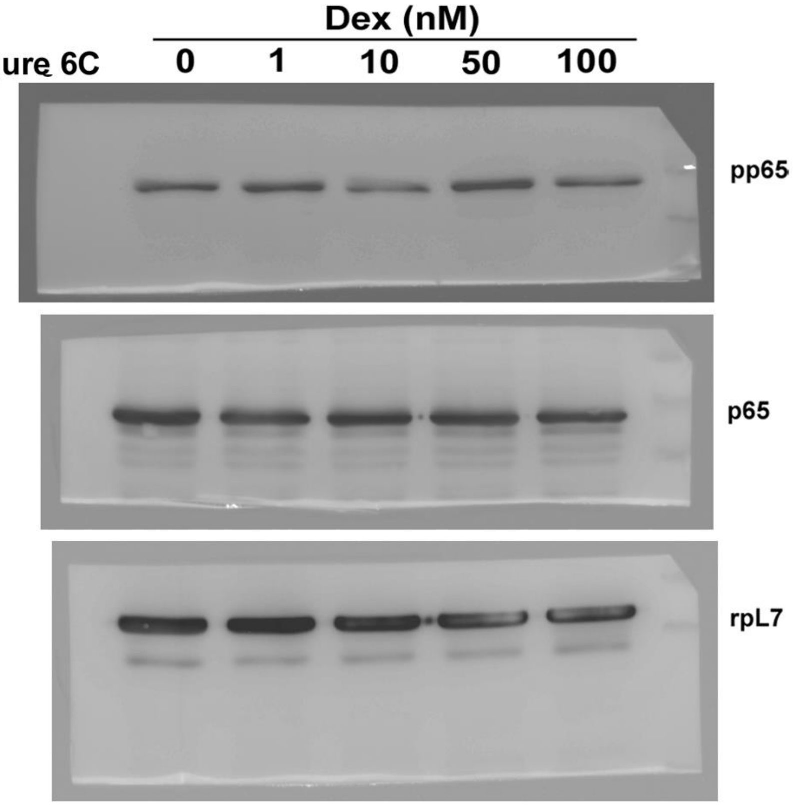

Figure 7A

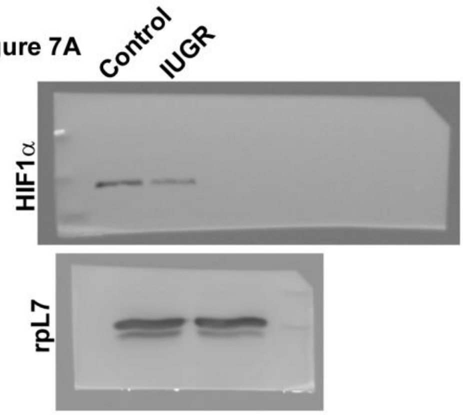


Figure 7C

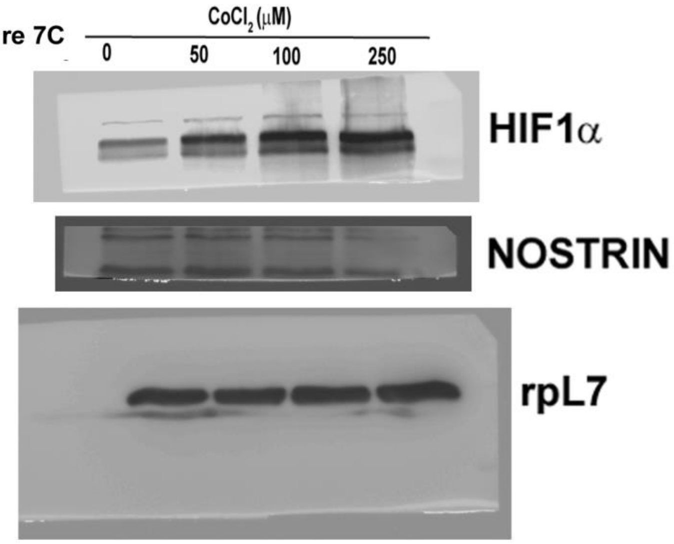


Figure 7F

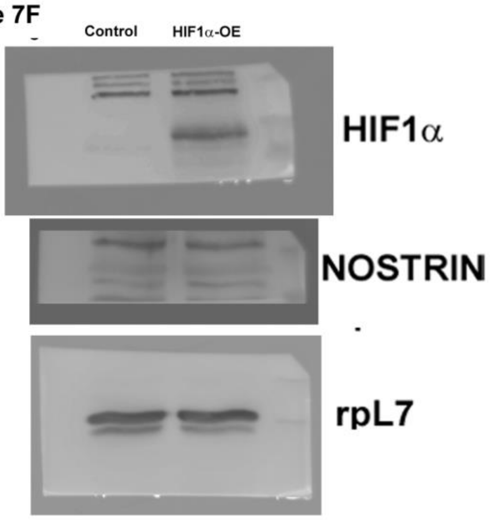

Supplement: Supplementary file 1 — Figure S1 and original blot imprints [file 41598_2018_26590_MOESM1_ESM.pdf]
